# Comparison of intensity, phase retardation, and local birefringence images for filtering blebs using polarization-sensitive optical coherence tomography

**DOI:** 10.1038/s41598-018-25884-w

**Published:** 2018-05-14

**Authors:** Shinichi Fukuda, Akari Fujita, Deepa Kasaragod, Simone Beheregaray, Yuta Ueno, Yoshiaki Yasuno, Tetsuro Oshika

**Affiliations:** 1 Department of Ophthalmology, Institute of Clinical Medicine, University of Tsukuba, Ibaraki, Japan; 2 Computational Optics and Ophthalmology Group, Ibaraki, Japan; 3 Computational Optics Group, University of Tsukuba, Ibaraki, Japan

## Abstract

Polarization-sensitive optical coherence tomography (PS-OCT) allows the recording of depth-resolved polarimetric measurements. It has been reported that phase retardation and local birefringence images can noninvasively detect fibrotic area in blebs after glaucoma surgery. Evaluation of scar fibrosis in blebs is important not only for predicting bleb function, but also for planning revision trabeculectomy. Herein, we characterize the intensity, phase retardation, and local birefringence images of blebs using PS-OCT. A total of 85 blebs from 85 patients who had undergone trabeculectomy were examined. Both phase retardation and local birefringence images detected fibrotic changes in blebs after glaucoma surgery. Phase retardation images detected slight fibrotic change during the early stage after surgery, whereas local birefringence images showed localized fibrotic tissue. There are two main patterns of local birefringence image changes in blebs: plate-like birefringence changes and diffuse changes. The area of plate-like birefringence change was significantly larger in poorly functioning blebs and is thus correlated with bleb function. These data suggest that the plate-like fibrotic change evaluation by PS-OCT may be useful not only for noninvasive evaluation of fibrotic scar tissue in blebs, but also for developing strategies for revision trabeculectomy.

## Introduction

The outcome of trabeculectomy, or *tube*-*shunt surgery*, depends on the formation of a functioning filtration bleb, which allows drainage of aqueous humor from the eye^1^. Moreover, the ability of the bleb to function determines the maintenance of the desired intraocular pressure (IOP)^2^. The most common reason for trabeculectomy failure is excessive scarring and fibrosis^3,4^. Postoperative subconjunctival scarring remains the major impediment to high success rates and often leads to poorly filtering blebs and a subsequent rise in IOP.

The use of antimetabolites such as 5-fluorouracil and mitomycin C to prevent conjunctival and episcleral fibrosis after trabeculectomy has been a major advance in glaucoma filtering surgery^5–7^. These antimetabolites have dramatically improved success rates in patients at high risk for trabeculectomy failure, thereby reducing the need for postoperative glaucoma medications. The use of these drugs, however, increases the risk of complications, such as bleb leaks, cataract, maculopathy, hypotony, and endophthalmitis^8^. If bleb fibrosis could be precisely evaluated, the dosage of the antimetabolite could be adjusted for each case.

Anti-inflammatory medication is also used to reduce the conjunctival scarring response^9^. Risk factors for bleb fibrotic scar-related failure include a long history of topical conjunctival medication use, conjunctival inflammation, previous surgical procedures breaching the conjunctiva, and younger age. If bleb fibrosis was recognized at an early postoperative stage, additional antifibrotic or anti-inflammatory treatment could be useful.

Filtration failure is regarded as the most frequent complication following trabeculectomy, which arises with time during the healing process and with the degree of fibrotic scarring. To maintain its filtering function, the bleb may need further medical treatment, including digital massage, removal of releasable sutures, laser suture lysis, repeat filtration surgery, or revision of the filtering site. Precise evaluation of fibrosis in a bleb would greatly assist ophthalmologists to develop individual strategies to refine these treatments.

Several studies evaluated fibrosis or blockage of fluid flow by conventional anterior segment optical coherence tomography (OCT), analyzing bleb morphology in a non-contact manner^10–13^. OCT is one of the most innovative and emerging optical imaging modalities during the past two decades. It has become a standard clinical tool in ophthalmology for non-invasive cross-sectional imaging as it shows not only retinal and choroidal structures^14^ but also anterior segment structures, such as the cornea and conjunctiva^10,11,15^. For instance, intensity images of functional blebs obtained by conventional anterior segment OCT reveal features such as large and tall blebs with internal hyporeflectivity and an increased number of microcysts^10–13^.

The polarization state of light changes when passing through abnormal fibrotic tissue (e.g., in the scar). Polarization-sensitive OCT (PS-OCT), an extension of conventional OCT, uses these polarization state changes to measure the depth-resolved polarization properties of postoperative fibrosis^16–22^. This feature makes PS-OCT a powerful technique for studying and potentially diagnosing postoperative fibrosis, especially in the bleb structure after glaucoma surgery. PS-OCT obtains two types of birefringence data, referred to as (1) phase retardation data and (2) local birefringence data^23^. PS-OCT can also obtain intensity images simultaneously, which perfectly co-register with the phase retardation and local birefringence images. Phase retardation images provide the accumulated polarization effect along the depth^16,18,19^. Local birefringence is a truly depth-resolved polarization quantity^20–22^.

We previously reported a high correlation between phase retardation alterations and bleb function in a large-scale study^18,19^. Phase retardation also allowed estimation of the prognostic value for the filtering bleb function during the early stage after glaucoma surgery^18^. Local birefringence imaging, in contrast, can detect the presence and location of highly birefringent tissue in the blebs^20–22^. No reports, however, have compared the characteristics of the intensity, phase retardation, and local birefringence images obtained with PS-OCT. In the current study, we therefore compared these characteristics using PS-OCT.

## Results

### Representative Images of Intensity, Phase Retardation, and Local Birefringence

**Figure 1:**
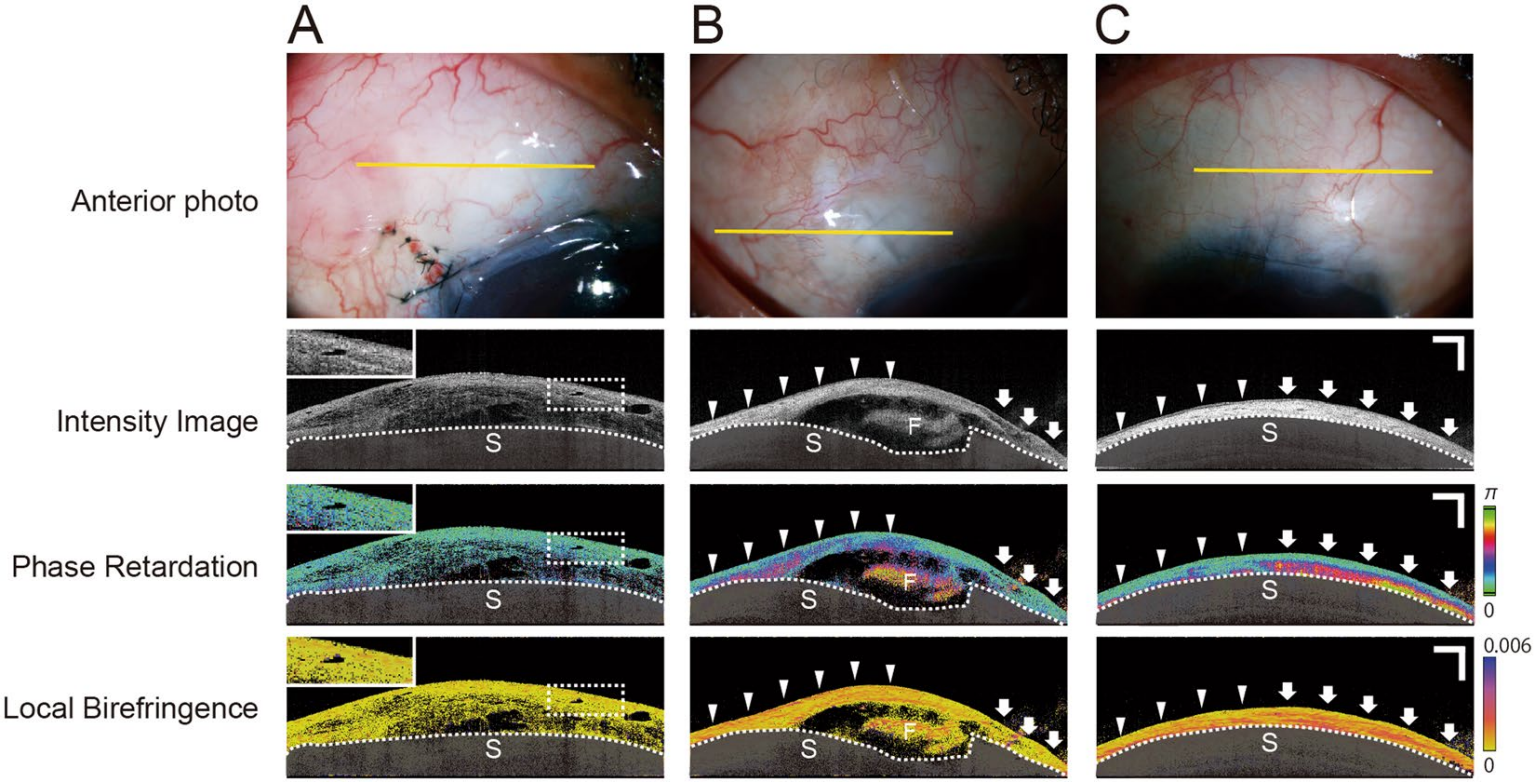
Representative intensity, phase retardation, and local birefringence images. (**A**) Good bleb function. An 80-year-old woman, 1 month after trabeculectomy, had intraocular pressure (IOP) of 9 mmHg without hypotensive medications. Sclera is covered with a semitransparent black shadow. The white dotted lines delineate the boundary between the conjunctiva and sclera. (**B**) A 65-year-old woman, 5 years after trabeculectomy, had IOP of 15 mmHg with hypotensive medications. High-reflectivity areas in the intensity image shows high phase retardation and local birefringence (arrowheads). Low reflectivity areas in the intensity image show low phase retardation and local birefringence (arrows). (**C**) Flat bleb. A 76-year-old woman, 3 years after trabeculectomy, had IOP of 15 mmHg with hypotensive medications. Note the hyperreflectivity, high phase retardation, and high birefringence areas (arrows) on the right, and the hyperreflectivity, mild phase retardation change and high birefringence areas (arrowheads) on the left. S = sclera, F = scleral flap. Scale Bars, 1 mm.

The representative cases in which intensity, phase retardation, and local birefringence images showed no fibrotic changes are shown in Fig. 1A. An 80-year-old woman, 1 month after trabeculectomy, had IOP of 9 mmHg without hypotensive medications (good bleb function). The bleb showed low birefringence in both the phase retardation and local birefringence images. The intensity image displayed a large low-reflectivity and many microcysts in the bleb wall (Fig. 1A). As co-located with the high-intensity region of the intensity image, slightly high birefringence was observed in the local birefringence image, although it was not detected in the phase retardation image (magnified inset in Fig. 1A).

A 65-year-old woman, 5 years after trabeculectomy, had IOP of 15 mmHg with hypotensive medications (poor bleb function). The filtration opening of the scleral flap could be seen. The bleb wall was thin and showed high reflectivity in the intensity image (Fig. 1B). The high-reflectivity area in the intensity image showed high phase retardation and local birefringence (Fig. 1B). In contrast, the low-reflectivity area in the intensity image showed low phase retardation and local birefringence (Fig. 1B).

A representative flat bleb is shown in Fig. 1C. A 76-year-old woman, 3 years after trabeculectomy, had IOP of 15 mmHg with hypotensive medications (poor bleb function). The entire conjunctiva showed high reflectivity, and there was no internal fluid shown in the intensity image. In contrast, the phase retardation and local birefringence images did not increase uniformly. They showed partial, strong fibrotic changes (Fig. 1C) that were not detected on the intensity image. The bleb displays stronger phase retardation and birefringence on the right side (Fig. 1C) in comparison to the left side (Fig. 1C).

### Local Birefringence Image Detection of Fibrotic Tissue

**Figure 2:**
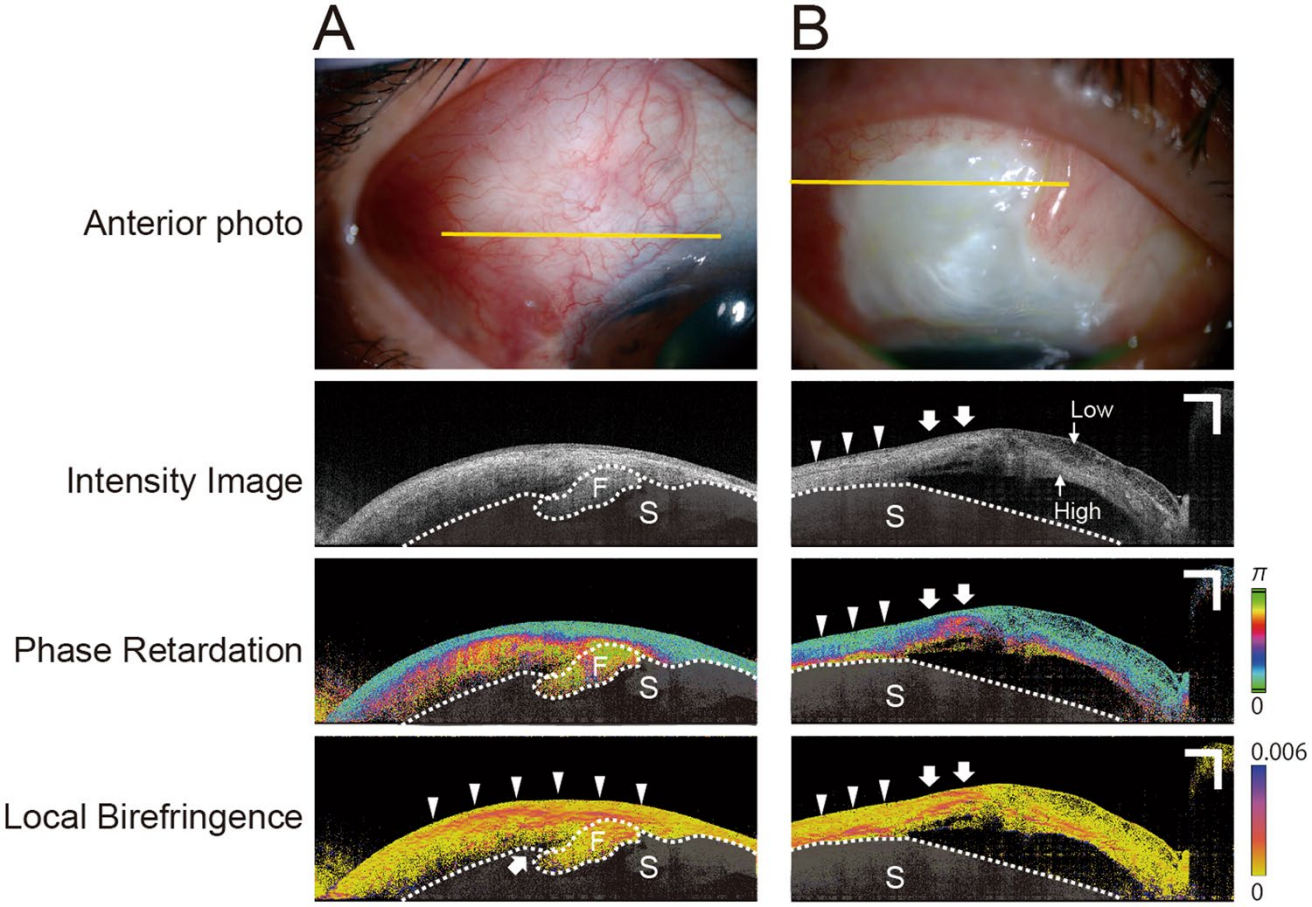
Representative cases show the sites of fibrotic tissue on local birefringence images. (**A**) A 57-year-old woman with a collapsed flat bleb, 4 months after trabeculectomy, had intraocular pressure (IOP) of 8 mmHg with hypotensive medications. Conjunctiva is not completely highly birefringent (arrowheads: covered by fibrotic tissue, arrows: open areas). (**B**) A 60-year-old man, 4 months after trabeculectomy, had IOP of 12 mmHg with hypotensive medications. The intensity image shows a low-reflectivity area (Low) and a high-reflectivity area (High), which are clearly separated from each other. The local birefringence image of the high-reflectivity area contains fibrotic tissue. The left area of the bleb shows high birefringence at the border of the conjunctiva and sclera (arrowheads), whereas the right area shows high birefringence in the middle of the conjunctiva (arrows). Sclera is covered with a semitransparent black shadow. The white dotted line delineates the boundary between the conjunctiva and sclera (S). Scale Bars, 1 mm.

The representative cases in which local birefringence images detected the location of fibrotic tissue are shown in Fig. 2. A 57-year-old woman with a collapsed, flat bleb, 4 months after trabeculectomy, had IOP of 8 mmHg with hypotensive medications (moderate bleb function). A large hyperreflective area but no microcysts were apparent in the intensity image. The phase retardation image revealed no fluid pool around the scleral flap, which was completely covered by high phase retardation conjunctiva. In contrast, high birefringence was not seen throughout the conjunctiva around the scleral flap (Fig. 2, arrowheads: covered by fibrotic tissue, arrows: open area). Only the local birefringence image detected fibrotic tissue uncovered area.

A 60-year-old man, 4 months after trabeculectomy, had IOP of 12 mmHg even with hypotensive medications (poor bleb function). There is a large avascular area in the bleb. The intensity image shows that the low-reflectivity and high-reflectivity areas were clearly separated from each other (Fig. 2B). The local birefringence image shows that the high-reflectivity area contained fibrotic tissue. The intensity and phase retardation images could not detect the precise location of fibrotic tissue (e.g., on the surface, middle, or border of the conjunctiva and/or sclera). The local birefringence image showed that the left area of the bleb had high birefringence at the border of scleroconjunctival border (Fig. 2B). In contrast, the right area on the bleb showed high birefringence at the middle of the conjunctiva (Fig. 2B). Only the local birefringence image recognized the location of the fibrotic tissue.

### Phase Retardation Image Detection of Early Fibrotic Change in the Bleb

We previously reported that phase retardation imaging detected early changes in fibrotic scar tissue and provided a prognostic value for filtering bleb function during the early stage after glaucoma surgery^18,19^. In the current study, nine cases were consecutively observed during the early postoperative period (mean 3.4 ± 1.2 months after surgery). Fibrotic changes become evident earlier in the phase retardation images than in the local birefringence images.

**Figure 3:**
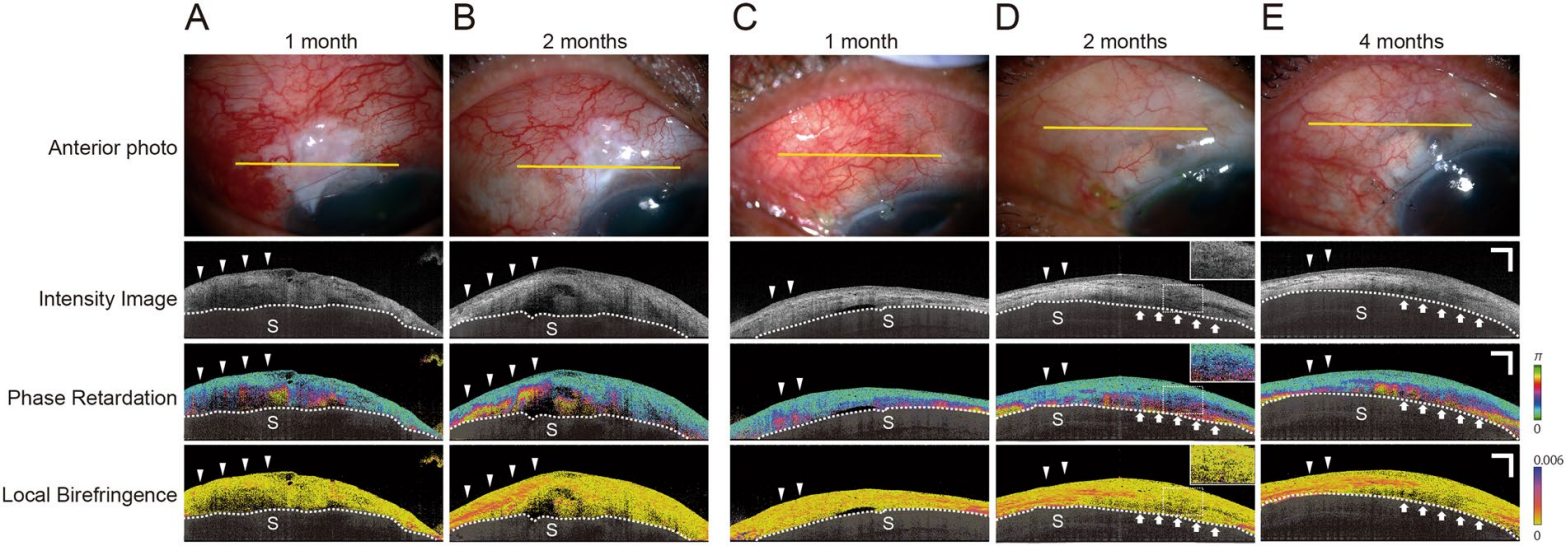
Representative cases in which phase retardation imaging detects early fibrotic changes in the bleb. (**A**,**B**) A 60-year-old man, 1 and 2 months after trabeculectomy (**A** and **B**, respectively, had intraocular pressures (IOPs) of 13 and 16 mmHg, respectively, without hypotensive medications. Most of the bleb shows low birefringence on a local birefringence image at 1 month (**A**), whereas there is an apparent increase in phase retardation on the phase retardation image (**A**, arrowheads). This area shows strong fibrotic changes on both phase retardation and local birefringence images at 2 months after surgery (**B**, arrowheads). (**C**–**E**) A 69-year-old man at 1 month (**C**), 2 months (**D**), and 4 months (**E**) after trabeculectomy. IOPs were 12 mmHg (1 month) and 17 mmHg (2 months) without hypotensive medications and 14 mmHg (4 months) with hypotensive medications. At 1 month after surgery, the intensity image shows mild hyperreflectivity. There is a mild increase in phase retardation and a slight diffuse change of birefringence. The area with the mildly increased phase retardation at 1 month shows strong fibrotic changes at 2 months after surgery (**C** and **D**, arrowheads). Arrows indicate the area of hyporeflectivity on the intensity image, as well as apparently increased phase retardation and low birefringence at 2 months after surgery (**D**, arrows). That area shows apparent degeneration of fibrotic change at 4 months after surgery (**E**, arrows). Sclera was covered with a semitransparent black shadow. The white dotted line delineates the boundary between the conjunctiva and sclera. Scale Bars, 1 mm.

Figure 3A,B show the bleb of a 60-year-old man, 1 month and 2 months after trabeculectomy, and IOPs of 13 and 16 mmHg, respectively, without hypotensive medications. The intensity image showed a combination of hyporeflective and mildly reflective areas. Multiple microcysts were found in the hyporeflective areas. Local birefringence imaging shows that most of the bleb had low birefringence. In contrast, phase retardation images showed increased phase retardation (Fig. 3A). Interestingly, at 2 months after surgery, both phase retardation and local birefringence images showed strong fibrotic changes in the area with increased phase retardation (Fig. 3B).

Figure 3C–E show the bleb of a 69-year-old man at 1, 2, and 4 months, respectively, after trabeculectomy. His IOPs were 12 and 17 mmHg (at 1 and 2 months, respectively) without hypotensive medications and 14 mmHg (at 4 months) with hypotensive medications. One month after surgery, the intensity image showed mild hyperreflectivity. Phase retardation was slightly increased. The local birefringence images showed a mild, diffuse increase in birefringence. The area with the slight increase in phase retardation at 1 month showed strong fibrotic changes in both phase retardation and local birefringence images at 2 months after surgery (Fig. 3C,D arrow head, respectively). Intensity imaging indicated an area of hyporeflectivity, increased phase retardation, and low birefringence at 2 months after surgery (Fig. 3D, arrow), as well as apparent deterioration of phase retardation at 4 months after surgery (Fig. 3E arrow). Importantly, bleb function became poor at 4 months after surgery. This slightly fibrotic change was difficult to judge based on the intensity or local birefringence images (Fig. 3D,E). Phase retardation images might be effective for evaluating mild fibrotic changes during the early stage after surgery, as previously reported. This finding is consistent with our previous report that phase retardation images provide an estimated prognostic value for filtering bleb function during the early stage after glaucoma surgery^18,19^.

### Detailed Analysis of Local Birefringence Images

**Figure 4:**
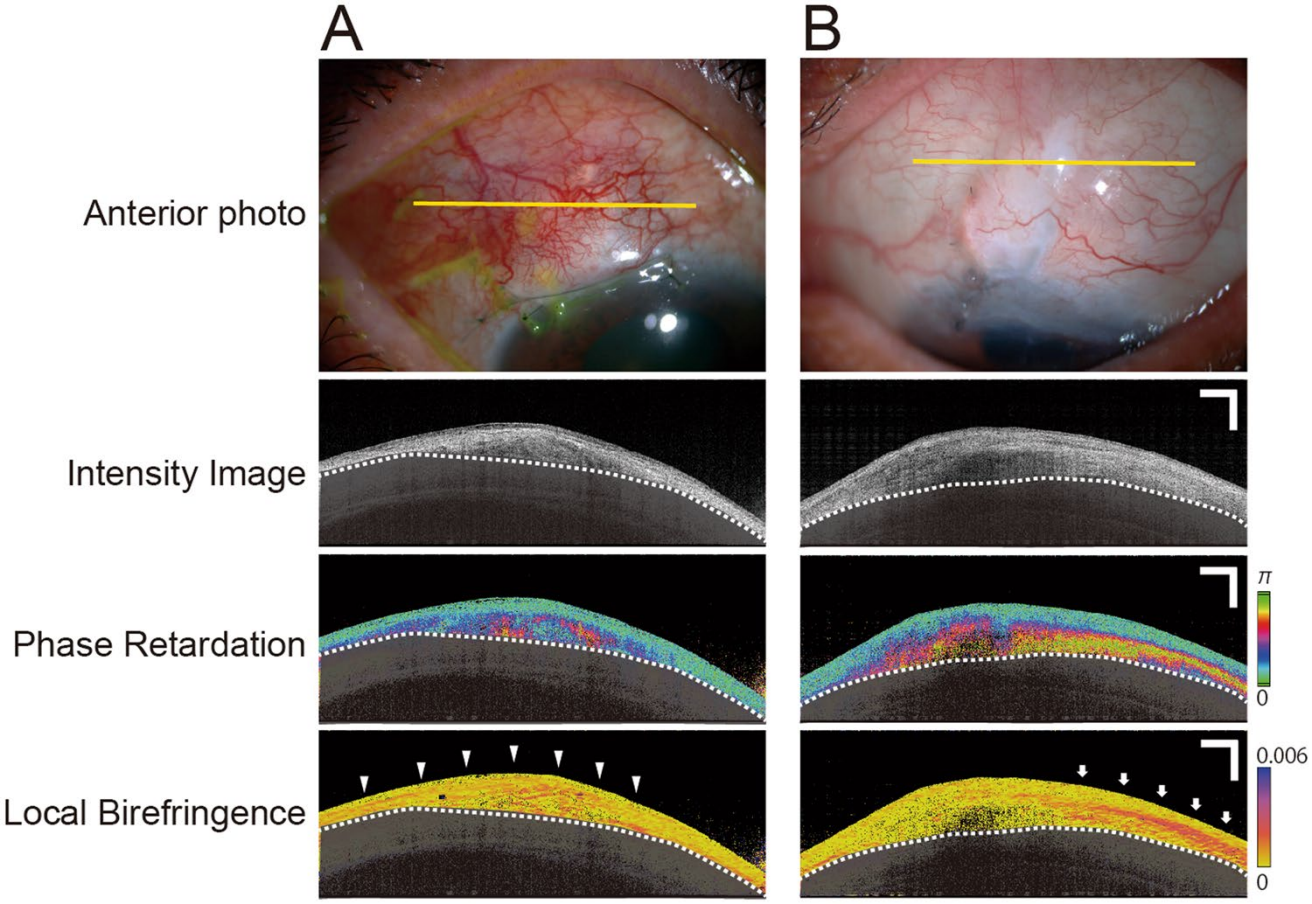
There are two main patterns of local birefringence in the bleb. (**A**) Diffuse change (arrowheads). (**B**) Plate-like birefringence change (arrows). Sclera is covered with a semitransparent black shadow. The white dotted line delineates the boundary between the conjunctiva and sclera. Scale Bars, 1 mm.

Two main patterns of local birefringence changes were observed in the blebs: diffuse changes (Fig. 4A) and plate-like birefringence changes (Fig. 4B). Local birefringence images detected the sites of plate-like birefringence changes. Many cases showed plate-like birefringence changes in the conjunctival bleb wall surrounding the fluid pool or the scleral flap. To investigate the influence of plate-like birefringence in bleb function, we evaluated the plate-like birefringence area located within four different minimum thresholds of bleb thickness. In other words, we measured the area of plate-like birefringence located within thicknesses of higher than 100 µm, 200 µm, 300 µm, and 400 µm.

**Figure 5:**
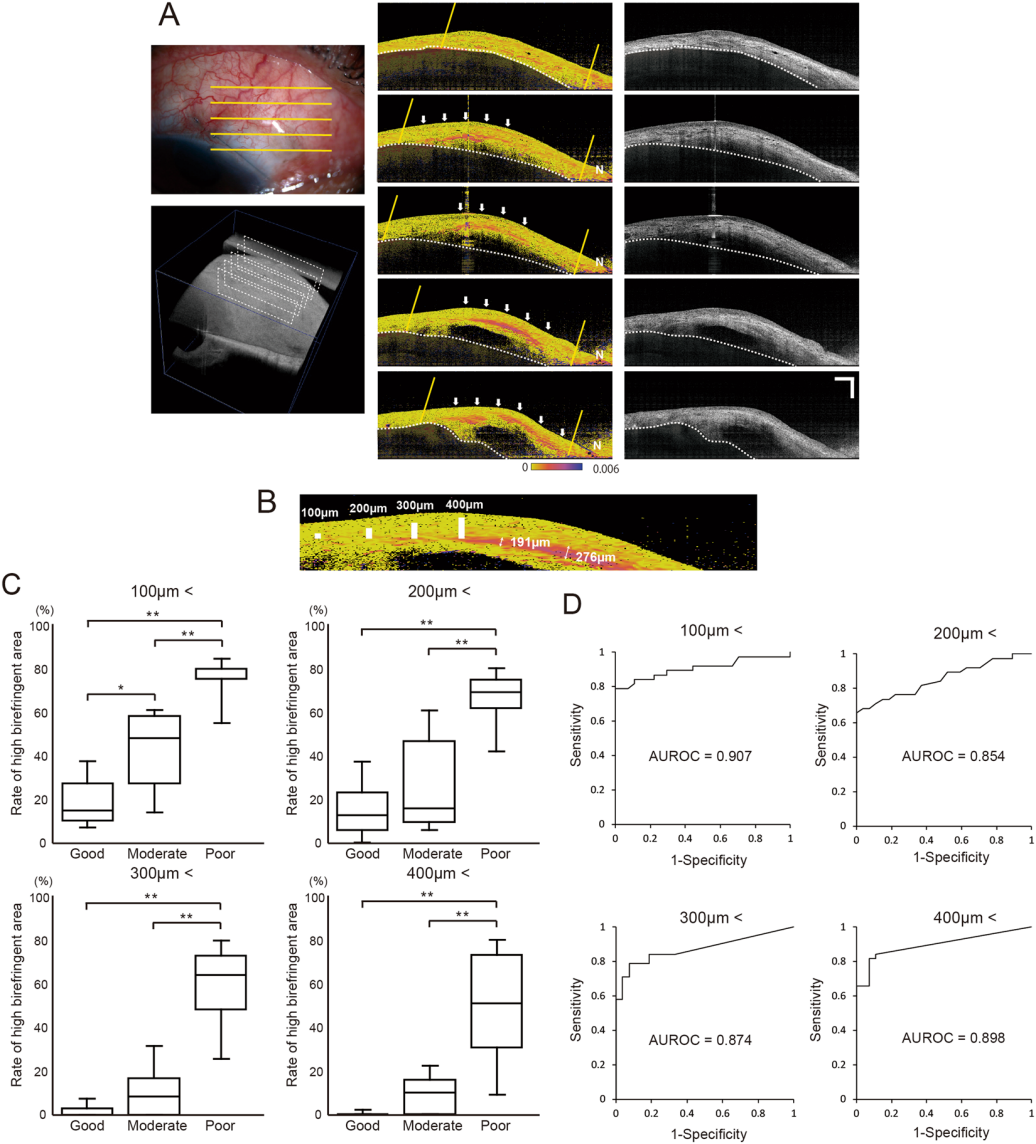
Relation between plate-like birefringence change and bleb function. (**A**) Five cross sections of local birefringence images are obtained and submitted to polarization-sensitive optical coherence tomography. The areas of the bleb and plate-like birefringence changes are measured. Thicken conjunctiva are defined as a bleb area. Strong signal noise area is excluded from bleb area (N). Final bleb area is comprehensively judged by using anterior photograph and cross section intensity images. The white dotted line delineates the boundary between the conjunctiva and sclera. Inside of two yellow line indicates bleb area. White arrow indicates plate-like fibrotic change. N: noise area. Sclera is covered with a semitransparent black shadow. Scale Bar, 1 mm. (**B**) Sample of thickness of plate-like fibrotic change. Scale bars: 100 µm, 200 µm, 300 µm and 400 µm. (**C**) Each definition of plate-like fibrotic change area as thicker than 100 µm, 200 µm, 300 µm, or 400 µm, the percent of area of plate-like birefringence change in regard to the whole bleb area. (*p < 0.005, **p < 0.0001, analysis of variance with the Bonferroni correction). (**D**) Each definition of plate-like fibrotic change area as thicker than 100 µm, 200 µm, 300 µm, or 400 µm, receiver operating characteristic (ROC) curve analyses for the comparison of the discrimination ability of bleb function. The “moderate” or “poor” bleb functions are considered as positive, and the “good” bleb function is considered as negative. The values of the area under receiver operating characteristic (AUROC) are also calculated.

Among the 85 blebs, 65 showed plate-like birefringence changes. The blebs with cystic and diffuse birefringence changes were excluded, leaving 27 good blebs, 15 moderate blebs, and 23 poor blebs for evaluation. The mean ages of the patients in each bleb group were 74.5 ± 11.1, 68.1 ± 10.6, and 70.0 ± 7.3 years, respectively. Their respective mean IOPs were 10.3 ± 2.9, 15.3 ± 1.2, and 15.2 ± 3.2 mmHg. The respective mean postoperative durations were 201.9 ± 419.3, 392.6 ± 826.2, and 1055.2 ± 1084.9 days. Representative plate-like birefringence change is shown in Fig. 5A. It was located around the internal fluid. The areas of plate-like birefringence change are shown in Fig. 5A,B.

When threshold of plate-like fibrotic change area was defined as thicker than 100 µm, there were significant differences among the bleb function between the good and moderate blebs (p = 0.0012), between the good and poor blebs (p < 0.0001), and also between the moderate and poor blebs (p < 0.0001), demonstrated by one-way analysis of variance (ANOVA) with Bonferroni correction. When plate-like fibrotic change area was defined as thicker than 200 µm, 300 µm, or 400 µm, there were significant differences between the good and poor blebs (p < 0.0001), and between the moderate and poor blebs (p < 0.0001). Therefore, according to the plate-fibrotic change area, good and moderate function blebs could only be distinguished when the cutoff value was higher than 100 µm (Fig. 5D). To determine the discrimination ability of bleb function, receiver operating characteristic (ROC) curve analyses were performed and the area under receiver operating characteristic (AUROC) were calculated. “Moderate” or “poor” bleb function was considered positive in the ROC analysis, while “good” bleb function was considered negative. The AUROC were 0.907, 0.854, 0.874 and 0.898 for definition of plate-like fibrotic change area thicker than 100 µm, 200 µm, 300 µm, or 400 µm (Fig. 5D).

There was a significant correlation between the area of plate-like birefringence change and bleb function (p < 0.0001, Spearman’s rank correlation).

## Discussion

OCT is a non-contact, non-invasive imaging technique that provides high-resolution, *in situ* visualization of bleb microstructure in real time. Several studies evaluated postoperative fibrosis in blebs according to their internal reflectivity using intensity images^10–12^. A large hyporeflective area was considered to represent a small amount fibrosis, which correlated with the function of the blebs^12^. The current study and previous studies revealed that the reflectivity on intensity images did not always correlate with tissue fibrosis^18,19,22^. We recently reported that PS-OCT was useful for evaluating bleb fibrosis after glaucoma filtration surgery using two types of birefringent imaging: phase retardation and local birefringence^18,19,22^. Phase retardation images show the accumulated polarization effect along the depth. Local birefringence images are from a depth-resolved polarization technique. PS-OCT can obtain intensity, phase retardation, and local birefringence images simultaneously. To the best of our knowledge, no previous report has compared these features.

It is known that phase retardation is performed using a relative delay of lights in two orthogonal eigen polarization states, with the data accumulating as light traverses a birefringent material. Thus, the phase retardation images obtained by PS-OCT accumulate from the tissue surface to the imaging point along the depth. We previously demonstrated a high correlation between altered phase retardation and bleb function^18^. Phase retardation values did not increase and bleb function was good until 2-weeks after glaucoma filtration surgery. At 1 month after surgery, the phase retardation value had significantly increased, although bleb function was still good. We also reported that the increase in phase retardation preceded the deterioration of bleb function, providing a prognostic metric for identifying changes in bleb function during the early stage after glaucoma surgery^19^. In the current study, phase retardation images detected a small change in fibrosis at an early postoperative stage that was not revealed in the intensity or local birefringence images. Interestingly, the same area developed fibrotic tissue, and the bleb function became progressively worse (Fig. 3), which is consistent with our previous reports^18,19^.

As phase retardation provides information on the accumulated polarization, it can detect even slight fibrotic changes. Automated detection of basal cell carcinoma (the most common skin cancer in humans) on intensity and phase retardation images obtained with PS-OCT yielded a classifier of high accuracy, sensitivity, and specificity^24^. Automated detection of the internal features of phase retardation might be useful in a detailed, precise analysis of fibrotic blebs.

The local birefringence images provided are capable of showing the precise location of scar tissue. Yamanari *et al*. evaluated local birefringence in blebs before and after needling revision and scar tissue removal^21^. In the current study, we found two typical types of local birefringence in blebs: diffuse change (Fig. 4A) and plate-like birefringence change (Fig. 4B). The area of plate-like birefringence change was significantly greater in poorly functioning blebs, and it correlated significantly with bleb function. Our results support those of previous reports that fibrotic scar tissue is involved in bleb function, and its removal may improve that function^21^. Excising fibrotic subconjunctival tissues is an important technique for bleb revision^21,25–29^. Yamanari *et al*. reported that, before bleb reconstruction, a narrow cleft in the filtering bleb and highly birefringent scar tissue were found at the site of partial adhesion of the scleral flap. They removed the scar tissue, with the area of the cleft becoming enlarged after needling revision and removing the scar tissue. Tsuda *et al*. reported the presence of highly birefringent tissue in the non-functional scarred bleb of human patients using PS-OCT and confirmed by Elastica–Masson staining and polarization microscopy^20^. The resected scarred bleb tissue showed high birefringence and PS-OCT examination after surgery showed that abnormal birefringence was reduced. It indicates that PS-OCT could accurately detect abnormally increased birefringence in human scarred bleb tissue.

Failure of the filtering procedure is a major problem after glaucoma surgery. When the IOP cannot be adequately controlled with medication, the management options are limited, and surgical intervention is often required. Bleb needling has been recognized as a relatively effective, simple approach to disrupting subconjunctival scar tissue and restoring bleb function. The initial bleb morphology was identified as a significant determinant of needling success. The central bleb area and height at the time of the needling procedure predicted survival^27,28^. A bleb with greater height predicted less scarring in the subconjunctival space and was thus a reasonable indicator of needling success. A smaller bleb with lower height also indicated the presence of outflow obstruction. Inadequate flow from the scleral flap area may result in more severe scarring. No reports have indicated that fibrosis of a bleb shown by PS-OCT is related to successful revision trabeculectomy.

It is difficult to formulate a clear strategy to ensure successful needling revision or repeated filtration surgery using intensity and phase retardation images because these images do not indicate the precise location of the scar tissue. In the current study, the local birefringence images revealed areas without fibrosis around the scleral flap (Fig. 2A). In the case shown in Fig. 1B, there was good bleb height and a filtration opening in the scleral flap. PS-OCT also showed that most of the bleb wall had fibrotic changes. Phase retardation and local birefringence images revealed an area of no fibrotic change. This information may be useful not only for developing a strategy for treating the bleb, but also deciding on the best approach when bleb revision surgery is required.

Bleb needling is used to disrupt subconjunctival fibrosis and episcleral scar tissue binding the scleral flap and subsequently to elevate the flap^28^. The current study revealed that plate-like birefringence change, which suggests strong focal fibrotic change, was widespread in some cases (Fig. 5A). The procedure might change, however, depending on the location and degree of fibrosis of the conjunctiva and around the scleral flap. For instance, needling a diffuse bleb with scarring may be less effective than needling a focal bleb with scarring^29^. Interestingly, when threshold of plate-like fibrotic change area was defined as thicker than 100 µm, plate-like fibrotic change area showed good discrimination ability between the good and moderate function blebs. On the other hand, threshold was defined as thicker than 200 µm, 300 µm, or 400 µm, plate-like fibrotic change area could not discriminate the good and moderate function blebs. As there were a few cases showing plate-like fibrotic change area thicker than 300 µm in the good and moderate function blebs, poor function blebs were easy to discriminate. These results indicate that plate-like fibrotic change was gradually became wider and thicker, and correlated bleb function. And it also suggests that if there are large area and thicker than 100 µm plate-like fibrotic change, it might be better to consider additional topical use of antimetabolites to prevent further fibrotic changes. Importantly, local birefringence image can indicate where to add these antimetabolites.

In the current study, no motion removal algorithm was used. However, internal bleb structures were assessed at least twice, and the best image was selected for subsequent analyses. The sparsity of the A-scans and B-scans does not negatively affect the phase retardation and birefringence measurements. Since the A-line and B-line spacing is similar or larger than the lateral optical resolution, the phase of OCT is not consistent among neighboring A-scans (or B-scans). This phase is a common phase term among the four entries of Jones matrix and is denoted as “global phase.” The global phase should be distinguished from the phases of each entries of the Jones matrix. This inconsistency of global phase disables complex averaging of OCT data among neighboring A-scans. The complex averaging had been used in some of our old studies, but it was not used in the present study. Although the global phase is not consistent, the mutual phase relations among Jones matrix entries are consistent within a domain in which the birefringence property are homogeneous. So we have applied “adaptive Jones matrix averaging method” to average the Jones matrix to enhance the signal-to-noise ratio. It ruled out the global phase first, and then average each entries of the Jones matrix^30^. The accuracy of birefringence measurement is further enhanced by applying maximum a-posteriori birefringence estimator. This estimator is also insensitive to the global phase, so it is compatible to the course lateral spacing. The characteristics of birefringence averaging and estimation and their compatibility to the coarse data spacing have also been summarized in a recently published review paper^31^. Although the spacing between B-lines is 5 times that the spacing between A-lines, image quality is not affected.

Our study had some limitations. The sample size was relatively small, and the observations were performed consecutively after surgery. We are planning a larger-scale study using consecutive observations after glaucoma surgery to evaluate the diagnostic ability of evaluating the combined intensity, phase retardation, and local birefringence images.

In conclusion, we have revealed the characteristics of intensity, phase retardation, and local birefringence images using PS-OCT after glaucoma surgery. Phase retardation images detected early fibrotic change. Local birefringence images detected plate-like fibrotic change and related it to bleb function. The plate-like fibrotic change was correlated to bleb function and may be useful for developing strategies for revision trabeculectomy.

## Methods

### Patients

A total of 85 blebs from 85 patients (54 men, 31 women; mean ± SD age 69.9 ± 10.4 years) who had undergone trabeculectomy were evaluated. Participants of this cross-sectional observational study were recruited at Tsukuba University Hospital from February 2013 to January 2015. The research conducted followed the tenets of the Declaration of Helsinki. Written informed consent was obtained from each participant. The institutional review board of Tsukuba University approved the study. Clinical trial registration was not required owing to the observational nature of the study. All participants were at least 18 years old.

### Trabeculectomy

Trabeculectomy was performed as reported previously^18^. Briefly, after a fornix-based conjunctival flap was prepared, a 4 mm wide half-layer square scleral flap was cut toward the clear cornea. The incision was made in the superior portion of the globe. A solution of 0.04% mitomycin C was prepared, and a surgical cellulose sponge soaked in the solution was held in contact with the area between the sclera and Tenon’s capsule for 3 min. The entire area was then irrigated with 200 ml of a balanced saline solution. The scleral flap was closed with four interrupted 10–0 nylon sutures. The conjunctiva was then closed tightly with interrupted 10–0 nylon sutures.

### PS-OCT

The PS-OCT images were obtained using a prototype engineered by the Computational Optics Group, University of Tsukuba, Tsukuba, Japan. In brief, the PS-OCT used in this study was based on Jones-matrix OCT, which uses a probe beam in two input polarization states. The system is based on swept-source OCT technology centered at a 1.3-µm wavelength with an operating speed of 30,000 A-scans per second. The axial and lateral resolutions in air were 12.7 and 20.5 µm, respectively.

Conventionally, a quantity called cumulative phase retardation was derived from the cumulative Jones matrix, which is the primary Jones matrix measured by PS-OCT. The phase retardation is a cumulative phase difference between two eigen polarization components of an incident light to a material, which is eye tissue in our case. It accumulates the polarization effect of the tissue as the light penetrates the tissue. So the phase retardation is a cumulative and not depth-localized polarization property of the tissue.

On the other hand, the birefringence is defined by the difference between two refractive indexes corresponding to the two eigen polarizations. Since the refractive indexes are depth-localized, the birefringence is also depth-localized. And hence, we use the term of “local birefringence” in this paper to emphasize its depth-localization nature.

In the PS-OCT, the phase retardation is obtained from the cumulative Jones matrix which is measured at a specific depth position from the surface of the tissue, and it accumulates the round trip polarization effect. It is noteworthy that the round trip polarization property is not always correctly related to a single trip polarization property of the tissue, which is our interest^23^.

In order to compute local birefringence, we first compute local Jones matrix within a small depth region from cumulative Jones matrix^32^ under an assumption that the local region is thin and homogeneous. This assumption is mostly correct in our case^23^. The local birefringence is finally computed from the local Jones matrix. The accuracy of the local birefringence measurement is further enhanced by using a maximum a-posteriori birefringence estimator^33,34^.

The resolution of the local birefringence image is defined by two factors. One is the size of the small depth region used to compute the local Jones matrix. It is 6 pixel (36.7 µm in depth) in our particular implementation. The other is the size of a small region, so called as kernel, of the maximum *a-posteriori* (MAP) birefringence estimator, where a birefringence value is estimated by using all the pixels in the kernel. In our implementation, the kernel size is 3 (lateral) ×3 (axial) pixels (70.2 µm ×18.4 µm in tissue). It should be noted that, although this process enables accurate quantification of local birefringence, it slightly degrades the spatial resolution because of the spatially extended operations.

The system was specifically designed to image the anterior eye segment. More details on this PS-OCT system are available in previous reports^17–19,22^.

The blebs were scanned using a horizontal, fast raster pattern. Scanning ranges of 12 × 12 mm, including 512 horizontal × 128 vertical A-lines, were obtained using PS-OCT. The data acquisition time of each volume was approximately 2.3 seconds.

In our previous cornea study using PS-OCT, we have investigated phase retardation of cornea on en face images. In the case of cornea, it was easy to automatically mark the boundaries between air and cornea/cornea and anterior chamber^35,36^. However, in the case of blebs, sometimes it is difficult to establish a clear boundary between bleb and sclera. Moreover; as the phase retardation represents cumulative information, the phase retardation images are influenced by the tissue thickness. The cornea did not have a large variation of thickness. On the other hand, there is a great variation in the bleb, such as flat bleb and huge encapsulated bleb. It does make interpretation of bleb en face images very difficult. Therefore, we decided to use cross section images for the current investigation. Internal bleb structures were assessed at least twice, and the best image was selected for subsequent analyses. Each PS-OCT image was measured by an experienced ophthalmologist (S.F.).

Intensity, phase retardation, and local birefringence images were obtained via PS-OCT. Approximate area of the bleb was selected by anterior photography (see Supplementary Fig. S1B). Anterior segment OCT (Casia SS-1000; Tomey, Nagoya, Japan) was measured simultaneously at every measurement for comparison between anterior photography and OCT cross section image. In anterior segment OCT, localization of En-face image and cross-section can be matched perfectly (see Supplementary Fig. S1A). We used blood vessel of En-face image as a landmark and manually compared with blood vessel of anterior photography (see Supplementary Fig. S1A). First, the boundary between the conjunctiva and the sclera was delineated based on the intensity image. The morphological boundary between the conjunctiva and the sclera was easily found as a dark space by intensity image (arrow head in see Supplementary Fig. S1B). Thickened conjunctiva were defined as a bleb area. Strong signal noise area is excluded from bleb area (Fig. 5A, see Supplementary Fig. S1B). Final bleb area was comprehensively judged by using anterior photograph and cross section intensity images. The intensity, phase retardation, and local birefringence images of PS-OCT measurement were obtained simultaneously, so they were perfectly co-registered.

### Area of Plate-like Fibrotic Change

We defined plate-like fibrotic change as a birefringence change of 200 µm observed on the local birefringence image. To investigate the influence of plate-like birefringence changes on bleb function, the area of plate-like birefringence change was evaluated. Altogether, 512 horizontal cross-sectional OCT images of the superior and inferior edges of the bleb were manually defined. Five cross-sectional local birefringence PS-OCT images were extracted at regular intervals. The boundary between the conjunctiva and sclera was delineated. The whole region of the conjunctiva in normal subjects presented no increase of phase retardation and local birefringence (see Supplementary Fig. S2). Sclera had very strong birefringence. Hence interpretation of phase retardation images of sclera is difficult for bleb cases which shows high phase retardation in the conjunctival regions. The sclera region was excluded from analysis. The cross-sectional bleb region was defined as a thickened conjunctival area anterior to the manually delineated scleroconjunctival boundary. To maximize the included bleb area, we also identified the bleb area by anterior photography. The apparent noise area on the PS-OCT image was excluded. The area of plate-like birefringence change was measured manually, and the percentage of the total bleb area that it represented was calculated (Fig. 5A).

The functionality of the bleb was classified based on the IOP and the patient’s hypotensive medications, as described in previous reports. In brief, bleb function was defined in three groups: “good,” “moderate,” and “poor.” A good bleb was defined as IOP ≤ 14 mmHg without hypotensive medication. A moderate bleb was defined as IOP ≥ 14 but ≤ 18 mmHg without glaucoma medication. A poor bleb was defined as IOP > 18 mmHg or if hypotensive medication had been prescribed to decrease IOP > 18 mmHg on two consecutive postoperative visits.

### Statistical Analysis

The relations between the area of plate-like local birefringence change and bleb function were analyzed using Spearman’s rank correlation test. A one-way analysis of variance (ANOVA) was performed to compare the areas of plate-like local birefringence change among the bleb function groups. All associations were considered statistically significant, with p < 0.05. The analyses were carried out using a commercial software package (StatView, version 5.0; SAS, Inc., Cary, NC). Each threshold definition of plate-like fibrotic change area (thicker than 100 µm, 200 µm, 300 µm or 400 µm), ROC curve analyses were performed to compare discrimination ability for bleb function. If bleb function was “moderate” or “poor”, it was considered positive for ROC analysis. If bleb function was “good”, it was considered negative for ROC analysis. The AUROC was also calculated. ROC analysis was performed using SPSS (version 19.0; IBM Corp., Armonk, NY).

## Electronic supplementary material

Supplementary information
