# Supplementary material for: Comparison of intensity, phase retardation, and local birefringence images for filtering blebs using polarization-sensitive optical coherence tomography
Source: Sci Rep. 2018 May 14;8:7519. doi: 10.1038/s41598-018-25884-w (PMC5951885; doi:10.1038/s41598-018-25884-w)
Supplement: Supplementary file 1 — Supplementary information [file 41598_2018_25884_MOESM1_ESM.pdf]

Supplementary information (Figures)

Comparison of intensity, phase retardation, and local birefringence images for filtering blebs using polarization-sensitive optical coherence tomography

Shinichi Fukuda,[1,2] Akari Fujita,[1] Deepa Kasaragod, [2,3] Simone Beheregaray,[1] Yuta Ueno,[1] Yoshiaki Yasuno,[2,3] Tetsuro Oshika [1,2]

[1]Department of Ophthalmology, Institute of Clinical Medicine, University of Tsukuba, Ibaraki, Japan
[2]Computational Optics and Ophthalmology Group, Ibaraki, Japan
[3]Computational Optics Group, University of Tsukuba, Ibaraki, Japan

Supplementary Figure S1

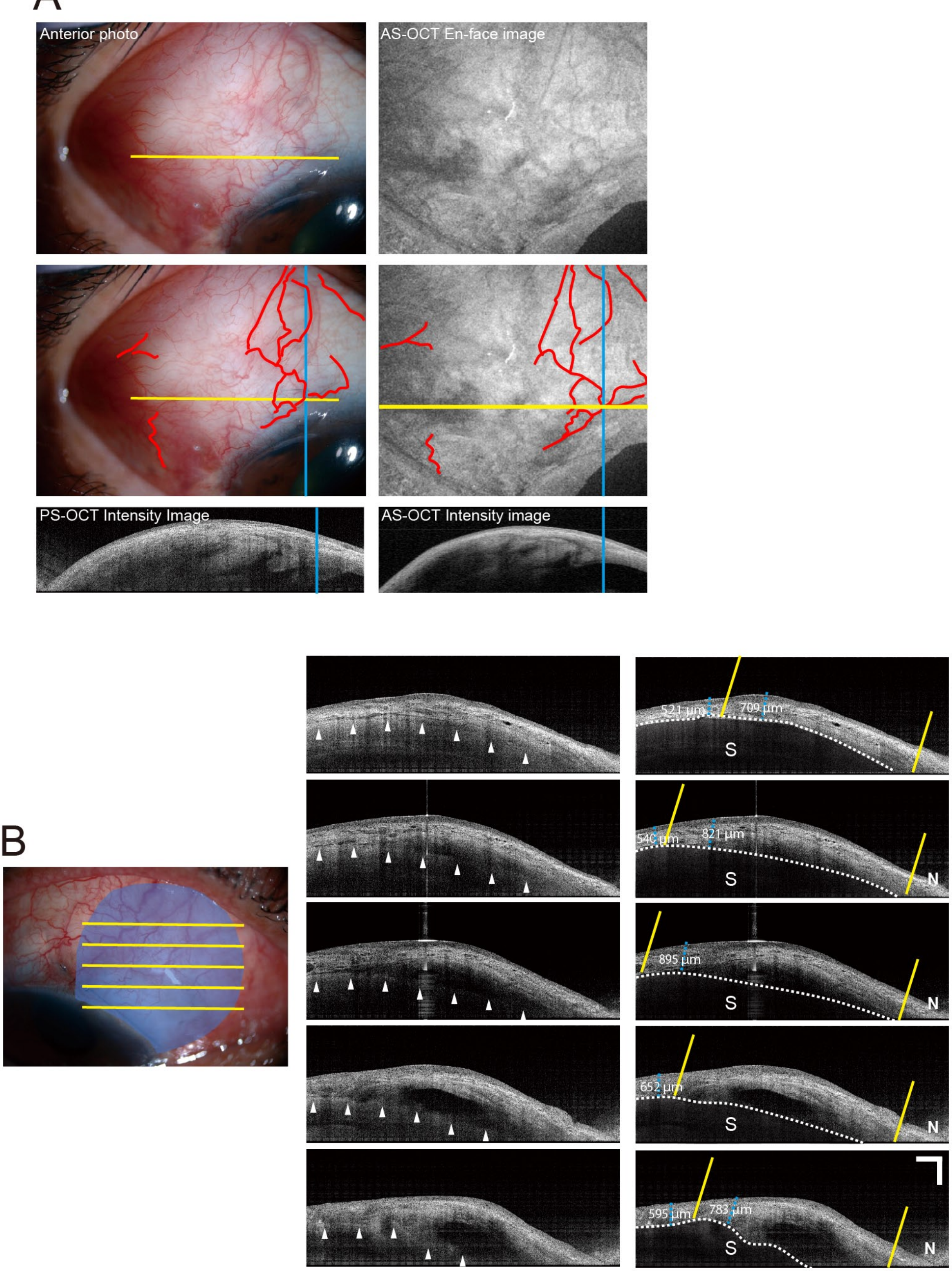

Supplementary Figure S2

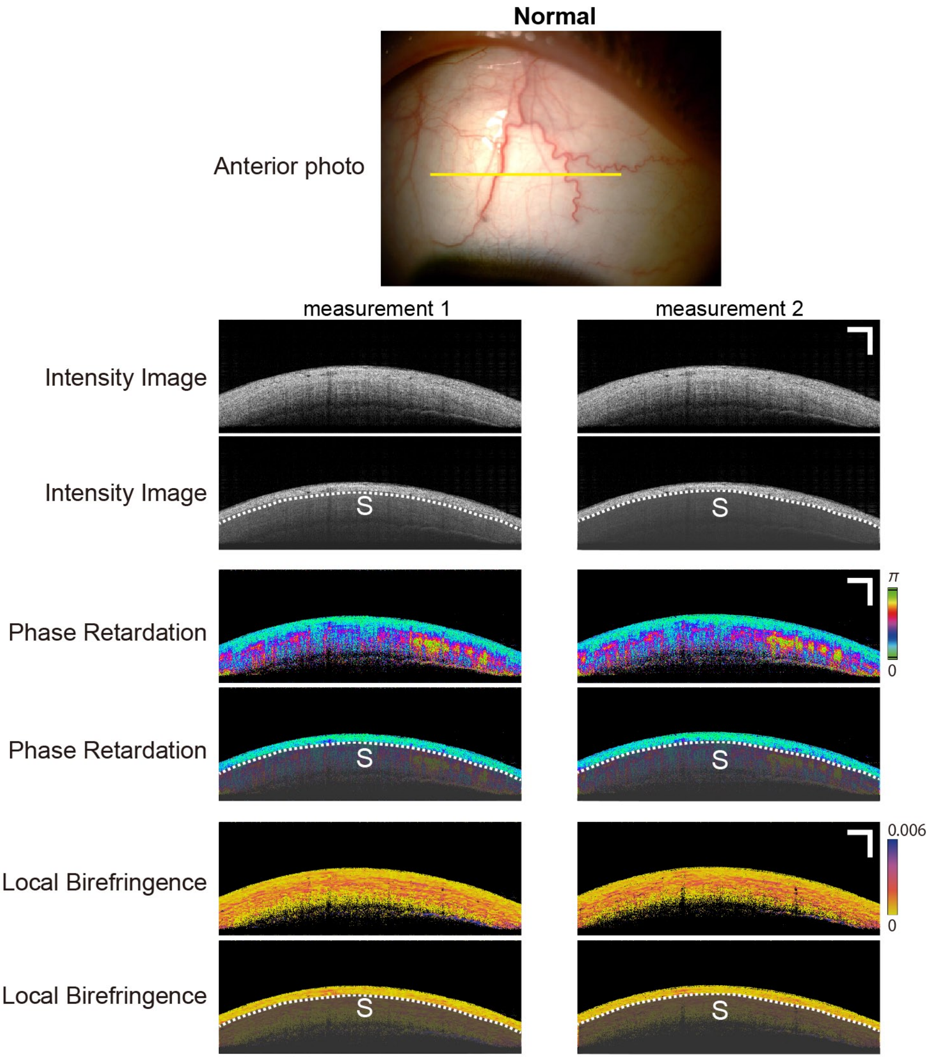

SUPPLEMENTARY FIGURE LEGENDS

Supplementary Figure S1. Comparison of anterior photography and anterior segment optical coherence tomography (AS-OCT) (**A**) Anterior photography and AS-OCT images. AS-OCT is measured simultaneously at every measurement for comparison between anterior photography and OCT cross section image. In AS-OCT, localization of En-face image and cross-section can be matched perfectly. Blood vessel manually segmentation on anterior photography and En-face image of AS-OCT. Blood vessel are used for landmark. (**B**) The approximate bleb area based on anterior photography. The morphological boundary between the conjunctiva and the sclera is easily found as a dark space (arrow head). Example of the thickness of conjunctiva. Bleb area shows thicker conjunctiva.

Supplementary Figure S2. Repeated intensity, phase retardation, and local birefringence images of normal subject. The white dotted line delineates the boundary between the conjunctiva and sclera. S = sclera. Scale Bars, 1mm.
